# AmPEP: Sequence-based prediction of antimicrobial peptides using distribution patterns of amino acid properties and random forest

**DOI:** 10.1038/s41598-018-19752-w

**Published:** 2018-01-26

**Authors:** Pratiti Bhadra, Jielu Yan, Jinyan Li, Simon Fong, Shirley W. I. Siu

**Affiliations:** Department of Computer and Information Science, University of Macau, Taipa, Macau China

## Abstract

Antimicrobial peptides (AMPs) are promising candidates in the fight against multidrug-resistant pathogens owing to AMPs’ broad range of activities and low toxicity. Nonetheless, identification of AMPs through wet-lab experiments is still expensive and time consuming. Here, we propose an accurate computational method for AMP prediction by the random forest algorithm. The prediction model is based on the distribution patterns of amino acid properties along the sequence. Using our collection of large and diverse sets of AMP and non-AMP data (3268 and 166791 sequences, respectively), we evaluated 19 random forest classifiers with different positive:negative data ratios by 10-fold cross-validation. Our optimal model, AmPEP with the 1:3 data ratio, showed high accuracy (96%), Matthew’s correlation coefficient (MCC) of 0.9, area under the receiver operating characteristic curve (AUC-ROC) of 0.99, and the Kappa statistic of 0.9. Descriptor analysis of AMP/non-AMP distributions by means of Pearson correlation coefficients revealed that reduced feature sets (from a full-featured set of 105 to a minimal-feature set of 23) can result in comparable performance in all respects except for some reductions in precision. Furthermore, AmPEP outperformed existing methods in terms of accuracy, MCC, and AUC-ROC when tested on benchmark datasets.

## Introduction

Antimicrobial peptides (AMPs) represent a large group of endogenous compounds widely distributed in nature. Owing to their broad spectrum of antimicrobial activities, such as antibacterial, anticancer, antiviral, antifungal, anti-inflammatory, and immunomodulatory properties, AMPs have become a model for developing novel antimicrobial drugs that may address the problem of increasing multidrug resistance of pathogenic microorganisms^1^.

In recent years, many computational methods were developed to accelerate the process of antimicrobial-drug discovery and design by providing a rational basis for candidate selection^2^. Machine learning algorithms have been the prime technique to train sequence-based classifiers to differentiate AMPs from non-AMPs. For example, prediction methods available on the CAMP website have been devised based on random forest (RF), support vector machine (SVM), artificial neural network (ANN), and discriminant analysis (DA) and have been trained on 257 features^3,4^. Multilevel classifier iAMP-2L was designed to predict AMPs and their activities by a fuzzy K-nearest neighbor algorithm and the pseudo–amino acid composition with 46 features^5^. A recently published SVM-based AMP classifier, iAMPpred, uses 66 features representing the computational, physicochemical, and structural properties of a peptide to predict its activity as antibacterial, antifungal, or antiviral^6^.

A crucial factor for the success of a prediction method is composition of the feature set. The ideal feature set should capture the major and subtle patterns from the sequence to differentiate actual positives from negatives. Our survey of existing methods^5–9^ has shown that compositional, physicochemical, and structural properties; sequence order; and the pattern of terminal residues are the most frequently adopted features for AMP predictions. By testing the predictive abilities of features in a systematic way, we have found that distribution patterns of amino acid properties including hydrophobicity, normalized van der Waals volume, polarity, polarizability, charge, secondary structure, and solvent accessibility are sufficient to identify AMPs with high accuracy. Distribution patterns of amino acid properties were first proposed by Dubchak *et al*. in 1995 as part of the Global Protein Sequence descriptors (composition-transition-distribution; CTD)^10^. CTD was developed for the protein-folding class prediction problem. Later, it was used for protein subcellular location prediction^11^ and protein function classification^12^. CTD was also included as part of the feature set for prediction methods provided on the CAMP web site^13^. Nevertheless, as feature selection was performed to reduce the feature set from 257 features to 64, it was not clear which selected features were in CAMP final models.

Here, we present AmPEP, a simple yet accurate AMP classification method based only on the distribution descriptors involving the RF algorithm. In an attempt to further reduce the feature set, we performed correlation analysis on the AMP/non-AMP distributions of each feature and showed that features with low AMP versus non-AMP correlations contributed to high accuracy of the prediction model. On the basis of this analysis, we generated a minimal set of 23 features that, to the best of our knowledge, is the smallest feature set for AMP prediction with high accuracy. Because the AMP dataset is highly imbalanced, we evaluated how different ratios of positives to negatives in the dataset can affect the predictive performance. To benchmark our method, at the end of the paper, we present a comparative analysis of our method against two other best-performing methods found in the literature: iAMPpred and iAMP-2L.

## Results

### Analysis of protein sequence descriptors

The CTD global protein sequence descriptors^10^ group amino acids into three classes for each physicochemical property. The complete CTD consists of three components: The Composition descriptor set (C) characterizes the global percentage of amino acids of each class in the sequence; the Transition descriptor set (T) characterizes the percent frequency of transitions between two classes along the sequence; and the Distribution descriptor set (D) characterizes the distribution patterns of amino acids of each class in the sequence. The three descriptor sets–C, T, and D—have been applied in various studies to other prediction problems^12,13^. Nevertheless, to determine whether CTD or each individual descriptor set alone is capable of AMP vs. non-AMP classification, we evaluated the predictive performance of four RF classifiers based on CTD, C, T, and D via 10-fold cross-validation by means of our collection of data M^model_train^. As shown in Table 1, the overall performance of the two smallest descriptor sets C and T is poorer than that of the other two with respect to accuracy (*Acc*), Matthew’s correlation coefficient (*MCC*), area under the receiver operating characteristic curve (*AUC-ROC*), and *Kappa* (*κ*). The model with the D descriptor set alone achieved almost similar performance relative to the model with CTD. These results indicate that among the three descriptor sets, D is capable of predicting AMPs with high accuracy but smaller complexity. Therefore, we adopted the Distribution descriptor set (abbreviated as D_F_ hereinafter) and performed its further analysis regarding AMP prediction.

**Table 1:** A comparison of four RF classifiers using different feature sets by 10-fold cross-validation with the AMP data ratio of 1:1. Values shown are the mean and standard deviation (in parentheses).

Feature set {#}	*Sn*	*Sp*	*Acc*	*MCC*	*AUC-ROC*	*AUC-PR*	*κ*
CTD {147}	0.979 (0.002)	0.944 (0.004)	0.961 (0.002)	0.924 (0.005)	0.988 (0.001)	0.698 (0.023)	0.923 (0.005)
C {21}	0.958 (0.002)	0.943 (0.004)	0.950 (0.002)	0.901 (0.004)	0.983 (0.001)	0.747 (0.018)	0.901 (0.004)
T {21}	0.959 (0.002)	0.943 (0.004)	0.951 (0.002)	0.901 (0.004)	0.983 (0.001)	0.745 (0.018)	0.901 (0.005)
D {105}	0.978 (0.002)	0.945 (0.004)	0.962 (0.002)	0.924 (0.004)	0.988 (0.001)	0.698 (0.024)	0.923 (0.005)

In total, D_F_ consists of 105 descriptors encoding distribution patterns of seven physicochemical properties of amino acids along the protein chain. Each descriptor is named after its *physicochemical property* (hydrophobicity, normalized van der Waals volume, polarity, polarizability, charge, secondary structure, or solvent accessibility), *class* (C1, C2, or C3), and *distribution type* (“first residue,” “25% residue,” “50% residue,” “75% residue,” or “100% residue” as 001, 025, 050, 075, or 100, respectively). We evaluated these descriptors on AMP vs. non-AMP classification by comparing the statistical distributions of the positive and negative samples in the M^model_train^ dataset and computed Pearson correlation coefficients (PCCs). A PCC that is close to zero indicates no correlation, ~0.3 denotes a weak correlation, and ~0.5 moderate correlation. Here, we are interested in testing whether these descriptors can help differentiate AMPs from non-AMPs, i.e., those that yield zero to moderate PCC values could be the most important descriptors for the classification task.

**Figure 1:**
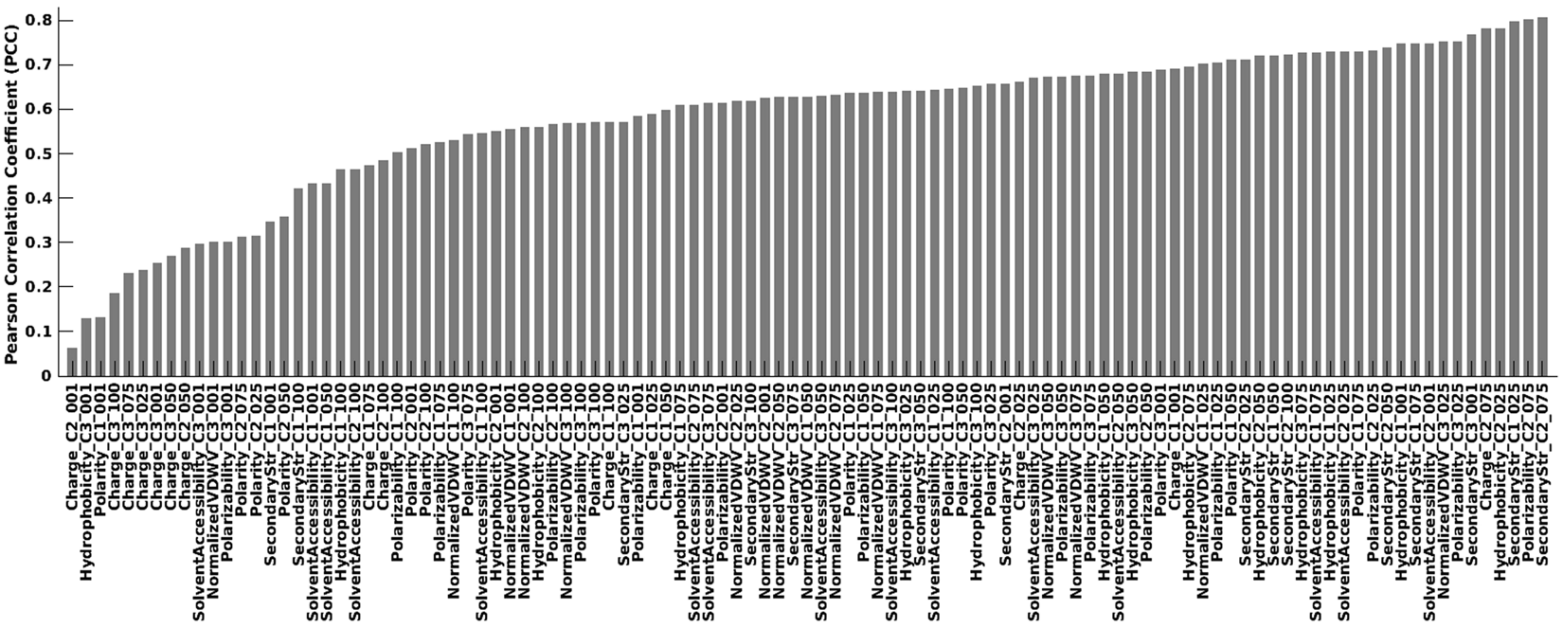
Pearson correlation coefficients (PCCs) between AMP and non-AMP distributions of the same descriptor in the M^model_train^ dataset.

As shown in Fig. 1 (also see Supplementary Table S1 and Figure S1), values of PCCs ranged from 0.06 to 0.81. Out of 105 descriptors, 23 (21.9%) had PCCs less than 0.5 (denoted as D_F_PCC<0.5_). Among them, nine, two, four, three, and five descriptors are of types first residue, 25% residue, 50% residue, 75% residue, and 100% residue, respectively. We can see that descriptors for first residue and 100% residue distribution types are among the most important descriptors. These descriptors encode physicochemical properties toward the N and C termini of the peptide chain. As summarized in a review by Bahar and Ren, peptide termini are crucial for its antimicrobial activity and resistance to proteases^14^. Because the average of D_F_PCC < 0.5_ of the first residue type of descriptors is 0.457, as compared to 0.540 for the 100% residue type, the N-terminal region of an AMP may contribute more than its C-terminal region to the antimicrobial activity.

To determine whether the same distribution patterns are present in other datasets, we performed PCC analysis on the benchmark datasets C^train^ and C^test^. In total, 15 descriptors were found to have PCC < 0.5 in all three datasets and are marked with asterisks in Supplementary Table S1. Looking closely again at the first residue and 100% residue descriptors, we observed that in most cases, the three datasets have similar average descriptor values. On the other hand, some large differences can be seen in 100% residue descriptors between C^train^ and the other two datasets as shown in Supplementary Figure S2. Although these two datasets contain different kinds of AMPs having a broad spectrum of activities such as antiviral, anticancer, antifungal, and anti-inflammatory, C^train^ contains only antibacterial peptides. Therefore, we presumed that these differences are important for the antibacterial activity of AMPs. Indeed, Lata *et al*.^7^ showed that there are differences in the terminal-residue profiles between antibacterial and nonantibacterial peptides. In addition to the C-terminal profile, Meher *et al*.^6^ suggested that composition, the net charge, isoelectric point, and propensity for secondary structure also differ among AMPs exerting different effects such as antibacterial, antiviral, and antifungal.

### Effects of AMP/non-AMP data ratios on prediction performance

Because experimental non-AMP data are scarce, a conventional way to produce negative samples is selection of random sequences from a protein database following certain criteria. This approach enables generation of a large amount of negative data. For training a classifier, one would usually select a positive/negative (P:N) data ratio of 1:1. By contrast, in a study by Li and coworkers on rebalancing data ratio techniques for inherently imbalanced medical data, they showed that in some cases, optimal classification accuracy and an optimal *κ* statistic can be achieved with a slight imbalance of the data distributions^15^. Here, we attempted to evaluate the effect of different P:N ratios on AMP prediction. From our own collection of data M^model_train^ (P:N ratio of 1:51), we generated 19 sets of data with P:N ratios ranging from 1:1, 1:1.5, and 1:2 up to 1:10. Because the amount of non-AMP data is large, for each P:N ratio, we constructed as many subsets of non-AMPs as possible by random selection without replacement. For example, at the P:N ratio of 1:1.5, the non-AMP data were sufficient to generate 34 different negative subsets. Details of the datasets for P:N ratio tests can be found in Supplementary Table S2. Then, we evaluated the predictive performance of each RF classifier with D_F_ features by 10-fold cross-validation and averaged the results across all subsets with the same P:N ratio.

**Figure 2:**
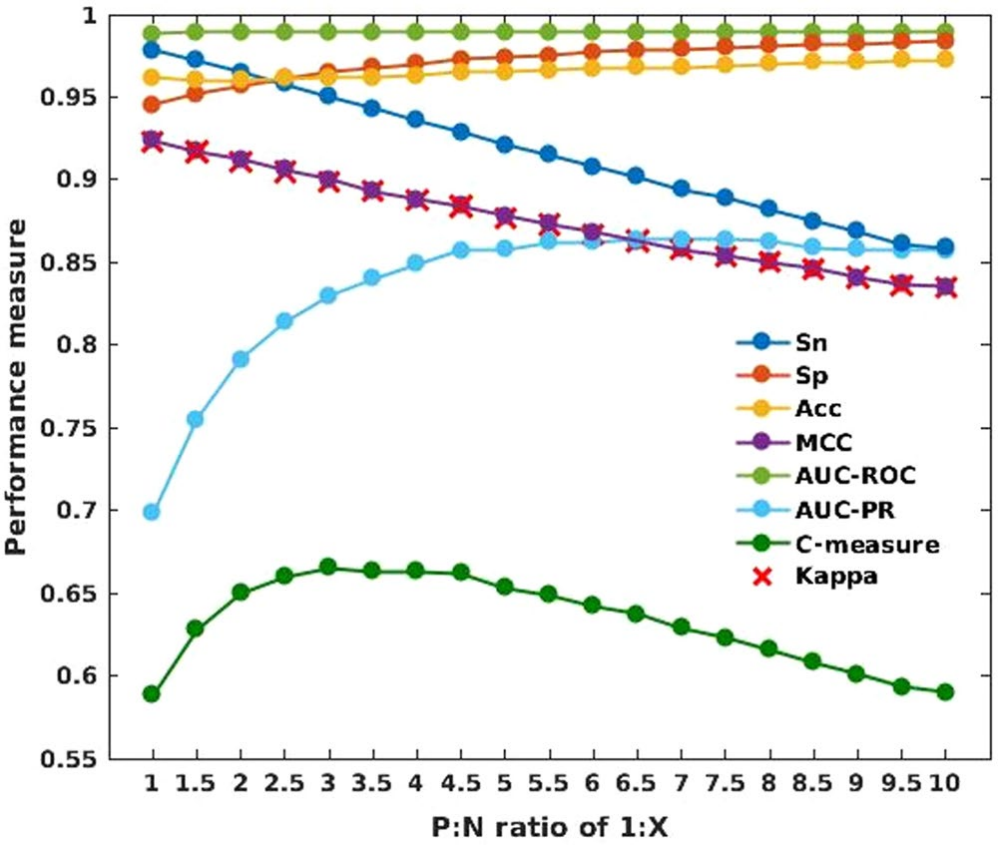
Performance of RF classifiers during 10-fold cross-validation on datasets with different AMP/non-AMP ratios.

Figure 2 presents the results on eight performance measures of the P:N ratio tests including sensitivity (*Sn*), specificity (*Sp*), *Acc*, *MCC*, *AUC-ROC*, area under the precision-recall curve (*AUC-PR*), *κ*, and *C-measure* (also see Supplementary Table S3 for all computed values). *C-measure* is a single metric combining four performance measures: *AUC-ROC*, *AUC-PR*, *MCC*, and *κ*. As shown in the figure, although *Sn*, *MCC*, and *κ* decrease as the dataset gets more imbalanced, *Sp* and *Acc* improve. This finding indicates that as the negative dataset gets bigger, the training procedure will inevitably switch its focus away from the positive samples (the minor class) and try to predict more correctly the negative samples (the major class). As mentioned in the Methods section, *AUC-ROC* is insensitive to the change in class distribution and more or less stays the same vis-Ã -vis different ratios of AMP/non-AMP data. In contrast to all the other measures where changes are linear, there is a drastic rise in *AUC-PR* as the amount of non-AMP data is increased, then it reaches a maximum at the P:N ratio of 1:6.5 and levels off at higher P:N ratios. Given that the amount of AMP data remains constant across these tests, the increase in *AUC-PR* (hence the precision) reveals that the reliability for positively predicted samples is greater when the amount of negative data is increased. A prediction model with high precision is particularly preferable for large-scale screening of AMPs in genome sequences where only highly reliable AMPs should be returned for the costly experimental validation.

To find an optimal model for general purposes, we selected *C-measure*, which combines the performance of four most popular metrics. According to *C-measure*, an RF classifier with the P:N ratio of 1:3 performs the best.

Recently, Synthetic Minority Over-sampling Technique (SMOTE) has been widely used as a preprocessing technique to rebalance the proportion of positive and negative samples before constructing the classifer. In SMOTE, in order to prevent information loss, instead of under-sampling the majority class it performs over-sampling by generating synthetic samples of the minority class from nearest neighbor samples^16^. To see if SMOTE improves AMP classification, we included SMOTE as part of the cross validation procedure where data in the training set is rebalanced by SMOTE and the constructed classifer is used to test the samples in the test set. As shown in Table 2, AmPEP methods with SMOTE perform comparably to AmPEP methods without SMOTE. While all four methods yielded very close accuracies, one can clearly see the shift in performance from higher sensitivity in AmPEP (1:1) to higher specificity in methods with larger proportion of initial negative samples. When the data is highly imbalanced (e.g. 1:10), SMOTE is likely to predict with biasing to the majority class, which is the negative class. Considering all major combined-measures (MCC, AUC-ROC, AUC-PR, Kappa), C-measure suggests that AmPEP (1:3) without SMOTE is the overall better option.

**Table 2:** Performance comparison of classifiers trained with P:N ratio of 1:1 and 1:3 against classifiers with applied SMOTE data rebalancing technique (*k* = 5) on initial data ratio of 1:3 and 1:10. Values shown were obtained from 10-fold cross validation.

Method	*Sn*	*Sp*	*Acc*	*MCC*	*AUC-ROC*	*AUC-PR*	*κ*	*C-measure*
AmPEP (1:1)	**0.978**	0.945	0.962	**0.924**	0.988	0.698	**0.923**	0.588
AmPEP (1:3)	0.950	0.965	0.962	0.900	0.989	0.830	0.899	**0.664**
AmPEP with SMOTE (1:3)	0.957	0.966	0.964	0.905	**0.990**	0.817	0.905	0.663
AmPEP with SMOTE (1:10)	0.858	**0.984**	**0.973**	0.835	**0.990**	**0.861**	0.835	0.594

### Descriptor selection

In an attempt to select the best descriptors among all 105, we evaluated the performance of models with reduced descriptor subsets. These subsets were generated based on the PCC analysis of AMP/non-AMP distributions. Three subsets–D_F_PCC<0.7_ (80 descriptors), D_F_PCC<0.6_ (43 descriptors), and D_F_PCC<0.5_ (23 descriptors)–were evaluated. As shown in Table 3, all three models with reduced descriptor sets maintained high accuracy as the full-feature model. Major differences are noticeable in *AUC-PR* and *C-measure*, in which performance is reduced by 4% and 5%, respectively, for D_F_PCC<0.6_ and by 6% and 7%, respectively, for D_F_PCC<0.5_.

**Table 3:** Performance of four RF classifiers involving different feature subsets during 10-fold cross-validation at the AMP/non-AMP data ratio of 1:3. Values shown are the mean and standard deviation (in parentheses). Each experiment uses all AMP data and a set of non-AMP data randomly drawn without replacement from the non-AMPs of the dataset. The best-performing models on a particular measure are highlighted.

Feature set {#}	*Sn*	*Sp*	*Acc*	*MCC*	*AUC-ROC*	*AUC-PR*	*κ*	*C-measure*
D_F_ {105}	**0.950** (0.003)	**0.965** (0.002)	**0.962** (0.002)	**0.900** (0.004)	**0.989** (0.000)	**0.830** (0.009)	**0.899** (0.004)	**0.665** (0.006)
DF_PCC<0.7 {80}	**0.950** (0.003)	**0.965** (0.002)	0.961 (0.002)	**0.900** (0.005)	**0.989** (0.001)	0.819 (0.011)	**0.899** (0.005)	**0.665** (0.010)
D_F_PCC<0.6_ {43}	**0.950** (0.003)	**0.965** (0.002)	0.961 (0.002)	0.899 (0.005)	**0.989** (0.000)	0.793 (0.008)	**0.899** (0.005)	0.633 (0.008)
DF_PCC<05 {23}	0.949 (0.004)	**0.965** (0.002)	0.961 (0.002)	0.898 (0.005)	0.988 (0.001)	0.779 (0.011)	0.898 (0.005)	0.620 (0.009)

### Comparative analysis of other variants of AAC, PAAC, and Covariance descriptors

Since amino acid composition (AAC) and pseudo amino acid composition (PAAC) have been widely used to predict various attributes of proteins^17^, the performance of RF classifiers using 10 variant descriptors of AAC, PAAC, and covariance were compared with AmPEP. Propy-1.0^18^ and Pse-in-One-1.0.4^19^ were used to calculate variant ACC, PAAC, and covariance protein descriptors from sequences. As shown in Table 4 for data ratio of 1:3, AmPEP using the full distribution descriptors and reduced descriptor set perform better than other RF classifiers using variant AAC and PAAC descriptors with respect to sensitivity, accuracy, *MCC*, *AUC-ROC*, and *κ*. It is interesting to note that AAC and PAAC perform only second to our methods in these aspects but they have improved specificity and *AUC-PR* while K-mer with the largest descriptor set among all methods yielded the highest specificity and *AUC-PR*. Similar performances were observed for classifiers using data ratio of 1:1 as presented in Supplementary Table S5.

**Table 4:** A comparison of RF classifiers using different descriptors by 10-fold cross-validation with the AMP data ratio of 1:3. Values shown are averages and standard deviations (in brackets) over 10 times of 10-fold cross validation.

Feature set {#}	*Sn*	*Sp*	*Acc*	*MCC*	*AUC-ROC*	*AUC-PR*	*κ*	*C-measure*
AmPEP {105}	**0.950** (0.003)	0.965 (0.002)	**0.962** (0.002)	**0.900** (0.004)	**0.989** (0.000)	0.830 (0.009)	**0.899** (0.004)	0.665 (0.006)
AmPEP {23}	**0.949** (0.004)	0.965 (0.002)	**0.961** (0.002)	**0.898** (0.005)	**0.988** (0.001)	0.779 (0.011)	**0.898** (0.005)	0.620 (0.009)
AAC {20}	0.910 (0.002)	**0.971** (0.000)	0.956 (0.001)	0.881 (0.002)	**0.989** (0.000)	0.862 (0.002)	0.881 (0.002)	0.662 (0.004)
PAAC {24}	0.910 (0.002)	0.970 (0.000)	0.955 (0.000)	0.881 (0.001)	0.985 (0.000)	0.891 (0.001)	0.881 (0.001)	0.681 (0.002)
K-mer {400}	0.898 (0.002)	**0.972** (0.000)	0.953 (0.001)	0.875 (0.002)	0.985 (0.000)	**0.918** (0.002)	0.875 (0.002)	**0.692 (0.003)**
Auto Covariance (AC) {6}	0.613 (0.003)	0.942 (0.001)	0.860 (0.001)	0.604 (0.003)	0.874 (0.001)	0.742 (0.003)	0.597 (0.003)	0.234 (0.003)
Cross Covariance (CC) {12}	0.661 (0.004)	0.949 (0.001)	0.877 (0.001)	0.657 (0.004)	0.905 (0.001)	0.769 (0.002)	0.651 (0.004)	0.298 (0.004)
Auto-Cross Covariance (ACC) {18}	0.710 (0.003)	0.951 (0.001)	0.891 (0.001)	0.698 (0.003)	0.922 (0.001)	0.825 (0.002)	0.695 (0.003)	0.369 (0.004)
Parallel Correlation Pseudo Amino Acid Composition (PC-PseAAC) {22}	0.908 (0.003)	**0.971** (0.001)	0.955 (0.001)	0.881 (0.002)	0.985 (0.000)	0.884 (0.005)	0.881 (0.002)	0.676 (0.006)
Series Correlation Pseudo Amino Acid Composition (SC-PseAAC) {26}	0.907 (0.002)	**0.971** (0.001)	0.955 (0.001)	0.880 (0.002)	0.985 (0.000)	0.882 (0.004)	0.880 (0.002)	0.673(0.005)
General Parallel Correlation Pseudo Amino Acid Composition (PC-PseAAC-General){22}	0.909 (0.002)	0.970 (0.000)	0.955 (0.001)	0.880 (0.002)	0.985 (0.000)	**0.896** (0.003)	0.880 (0.002)	**0.683 (0.003)**
Parallel Series Correlation Pseudo Amino Acid Composition (SC-PseAAC-General) {26}	0.908 (0.002)	0.970 (0.001)	0.955 (0.001)	0.879 (0.002)	0.985 (0.000)	0.894 (0.003)	0.879 (0.002)	0.680 (0.003)

The best two results in each performance measure are highlighted. AAC: Amino Acid Composition, PAAC: Pseudo Amino Acid Composition AAC and PseAAC were generated using propy 1.0 package (default parameter of propy is used). Other descriptors, K-mer, AC, CC, ACC, PC-PseAAC, SC-PseAAC, PC-PseAAC-General, SC-PseAAC-General were generated by Pse-in-One-1.0.4 using default parameters.

### Comparative analysis with state-of-the-art methods

To further assess the predictive ability of our AMP prediction method, we trained the RF models of DF, D_F_PCC<0.7_, D_F_PCC<0.6_, and D_F_PCC<0.5_ on C^train^ and compared their performance on C^test^ against two latest AMP prediction methods, namely, iAMPpred^6^ and iAMP-2L^5^. The reason why only two methods were included here for comparison is that iAMPpred and iAMP-2L were recently shown to be the best two approaches among eight AMP prediction methods on the same benchmark datasets^6^. As shown in Table 5, our model AmPEP (DF) outperforms these two methods with respect to *AUC-ROC* and *MCC* by 2–5% and 1–2%, respectively, though *AUC-PR* is reduced by 3% as compared to iAMPpred. It is worth noting that our models with reduced descriptor sets also perform similarly to the existing methods with a similar feature set size.

**Table 5:** A comparison of our AMP prediction method with state-of-the-art methods on AUC-ROC, AUC-PR, MCC, and *κ* by means of datasets C^train^ and C^test^.

Method	ML algorithm	Number of features	AUC-ROC	AUC-PR	MCC	*κ*
iAMPpred#	SVM	66	0.98	**0.99**	0.91	—
iAMP-2L#	FKNN	46	0.95	—	0.9	—
AmPEP (D_F_)	RF	105	**0.995**	0.957	**0.920**	0.962
AmPEP (D_F_PCC < 0.7_)	RF	80	0.994	0.950	0.914	0.913
AmPEP (D_F_PCC < 0.6_)	RF	43	0.994	0.934	0.919	0.918
AmPEP (D_F_PCC < 0.5_)	RF	23	**0.995**	0.905	**0.924**	0.923

^#^Results were taken from refs^5,6^.

## Discussion

A serious public health problem is the failure of conventional antibiotics to kill pathogenic bacteria because of the development of multidrug resistance. Computational methods that can quickly and accurately identify candidate peptides as AMPs for subsequent experimental assays are necessary to shorten the drug discovery process. To this end, we developed a highly accurate sequence-based AMP classification method, named AmPEP, using distribution patterns of amino acid properties. Our feature set is composed of 105 distribution descriptors covering seven physicochemical properties of peptides (hydrophobicity, normalized van der Waals volume, polarity, polarizability, charge, secondary structure, and solvent accessibility). For each property, the class distribution pattern was characterized on the basis of the sequence as a position percentage: the first residue, 25% residue, 50% residue, 75% residue, and 100% residue type of distribution.

Although there are a few AMP prediction methods available, the development of AmPEP is different from that of the existing approaches in several ways: First of all, distribution descriptors were for the first time used alone as features for identification of AMPs, whereas all the existing AMP prediction methods employ a combination of features such as composition of amino acids and pseudo–amino acid code. Furthermore, to the best of our knowledge, our work contributed to the construction of the largest and diverse AMP dataset for the purpose of machine learning model evaluation. Our positive data were curated after retrieval from three major databases (CAMP, APD3, and LAMP), whereas most of the earlier methods involve a single database: either CAMP or APD^5,20,21^. Because experimental negative data are scarce, the negative data here were generated from UniProt sequences. Instead of restricting the sequence length to 100 amino acid residues or less as other methods do^5,20^, we included protein sequences 5–255 residues long to cover the same length space as in our collection of known AMPs. The final dataset for model construction contains 3,268 AMPs and 166,791 non-AMPs. The large amount of non-AMP data allowed us to assess, for the first time, the prediction model performance at different positive-to-negative data ratios. A total of 19 data ratios were tested on eight performance measures. Our proposed combined metric, *C-measure*, takes into account four performance characteristics (*MCC*, *AUC-ROC*, *AUC-PR*, and *κ*) when selecting an optimal model with accuracy, precision, and credibility.

The ultimate goal of AMP prediction is to design new peptide sequences with desirable antimicrobial and therapeutic effects. For example, on the basis of frequently occurring residue information from the ADP database, Wang *et al*. successfully designed a short AMP, GLK-19, with higher activity against *Escherichia coli* relative to human AMP LL-37^22^. Although peptide design is outside the scope of this paper, we believe that an understanding of the role of residues, their properties, and positions in the sequence will be crucial for the design of an AMP *de novo*. As a first step toward acquisition of this information, we performed analyses of AMP and non-AMP distributions of all the distribution descriptors. By means of PCCs, we were able to rank descriptors that can better differentiate AMPs from non-AMPs. Based on our analysis, *Charge* was found to be the key factor for antimicrobial activity. We also found that properties in terminal regions are significantly different between the two classes, in agreement with experiments. According to these analyses, we believe that the distribution patterns of properties learned from AMP and non-AMP sequences can be applied to establish helpful guidelines for the *de novo* design of highly active AMPs.

The performance of AmPEP was compared with other prediction methods on benchmark datasets. AmPEP was found to have higher *AUC-ROC* and *MCC* than iAMPpred and iAMP-2L do. We showed that with a reduced number of features (from 105 to 23), AmPEP can still show comparable performance. At present, the 23-feature set is the smallest feature set for AMP prediction by the machine learning approach.

Proposed AmPEP is an attempt to develop a highly accurate RF classifier for AMP prediction based on distribution patterns of physicochemical properties. The distribution patterns are believed to facilitate *de novo* AMP design, and AmPEP can address the time and cost limitations of experimental processes for designing a novel AMP. In view of the success of ensemble learning for AMP prediction, our future work will explore more recent ensemble learning techniques such as selective ensemble for training the classifier^23,24^. Our current method relies on PCCs to select important features for the final classifier. We believe that an advanced and intelligent feature selection strategy such as that in refs.^25,26^ may yield an improved model because noisy features will be eliminated.

## Methods

### The training dataset for model construction

We generated our own positive dataset by retrieving naturally occurring and experimentally validated AMP sequences from major databases, namely, APD3^27^, CAMPR3^4^, and LAMP^28^, which contained 2338, 5549, and 3050 sequences, respectively. We pooled all the AMPs and removed duplicated ones; sequences with unnatural amino acids (B, J, O, U, X, and Z) were also eliminated to form a nonredundant dataset.

Given that experimentally validated negative AMP sequences are rarely found in the literature, we followed the data preparation procedure undertaken in other studies^5,20^ to generate a negative dataset. First, all protein sequences 5 to 255 amino acid residues long were downloaded from UniProt. Then, we removed all sequences that were annotated as AMP, membrane, toxic, secretory, defensive, antibiotic, anticancer, antiviral, and antifungal. Unique sequences were extracted, and sequences with unnatural amino acids (B, J, O, U, X, and Z) were removed. The final training dataset for model construction, M^model_train^, contains 3268 AMPs and 166791 non-AMPs.

Our datasets are freely accessible on the CBBio homepage at http://cbbio.cis.umac.mo/software/AmPEP/.

### Benchmark datasets for comparative analysis

The performance of our method was compared with that of the latest AMP prediction methods. The comparison was made by means of the benchmark dataset of Xiao *et al*.^5^. The same dataset was used in a recent study by Meher *et al*. for a comparison of AMP prediction methods^6^. Overall, the training set (C^train^) contains 770 AMPs and 2405 non-AMPs. The test set (C^test^) contains 920 AMPs and 920 non-AMPs.

All the datasets used in this study are summarized in Table 6.

**Table 6:** A summary of the positive and negative datasets. Values in braces are the numbers of sequences collected in that category.

Dataset	Model Design	Comparative Study	
	Training (M^model_train^)	Benchmark Training (C^train^)	Benchmark Testing (C^test^)
Positive	APD3, CAMPR3, LAMP {3268}	Xiao {770}	Xiao {920}
Negative	UniProt {166791}	Xiao {2405}	Xiao {920}

### Features

The goal of machine learning is to learn the feature pattern from data points and to find optimal model parameters that yield the highest accuracy while avoiding the problem of overfitting. Data points are converted into features also known as *descriptors*. To identify AMPs by means of sequences alone, we have to convert the plain amino acid sequences into numerical descriptors characterizing different properties of peptides. In this study, we employed the *Distribution* (DF) descriptor set from the Global Protein Sequence Descriptors proposed by Dubchak *et al*.^10^ originally for protein-folding class prediction. It encodes distribution patterns of physicochemical properties of amino acids along the protein chain. As shown in Table 7, seven physicochemical properties are considered: (1) hydrophobicity, (2) normalized van der Waals volume, (3) polarity, (4) polarizability, (5) charge, (6) secondary structure, and (7) solvent accessibility. For each of these seven properties, amino acids are grouped into three classes. For example, for the hydrophobicity property, the three classes are *Polar* (R, K, E, D, Q, and N), *Neutral* (G, A, S, T, P, H, and Y), and *Hydrophobic* (C, L, V, I, M, F, and W). For each of the 21 (=7×3) classes, five *distribution* descriptors representing the position percentage–the first residue, 25% residue, 50% residue, 75% residue, and 100% residue distribution type–of a particular class in a sequence are computed. The “first residue” of a particular class is the first occurrence of any of the class residues along the sequence. The descriptor value is calculated as the percentage of residues before and include this residue from the N terminus. For the “Y% residue” type of distribution of a particular class, we first compute $Z=\lfloor R\times Y/100\rfloor$, where *R* is the total number of class residues in the sequence, and *Y* denotes the desired percentage. The position of the Zth occurrence of the class residues, denoted as *P*, can be determined from the sequence, then the descriptor value of the “Y% residue” distribution type of a particular class is finally computed as (*P*/*L*) × 100%, where *L* is the total length of the sequence. The descriptor value is 0 if none of the class residues is present in the sequence.

**Table 7:** Physicochemical properties and groupings of amino acids.

Property	Grouping		
	Class 1 (C1)	Class 2 (C2)	Class 3 (C3)
Hydrophobicity	Polar R, K, E, D, Q, N	Neutral G, A, S, T, P, H, Y	Hydrophobic C, L, V, I, M, F, W
Normalized van der Waals volume	Volume range 0-2.78 G, A, S, T, P, D	Volume range 2.95-4.0N, V, E, Q, I, L	Volume range 4.03-8.08 M, H, K, F, R, Y, W
Polarity	Polarity value 4.9-6.2 L, I, F, W, C, M, V, Y	Polarity value 8.0-9.2 P, A, T, G, S	Polarity value 10.4-13 H, Q, R, K, N, E, D
Polarizability	Polarizability value 0-0.108 G, A, S, D, T	Polarizability value 0.128-0.186 C, P, N, V, E, Q, I, L	Polarizability value 0.219-0.409 K, M, H, F, R, Y, W
Charge	Positive K, R	Neutral A, N, C, Q, G, H, I, L, M, F, P, S, T, W, Y, V	Negative D, E
Secondary structure	Helix E, A, L, M, Q, K, R, H	Strand V, I, Y, C, W, F, T	Coil G, N, P, S, D
Solvent accessibility	Buried A, L, F, C, G, I, V, W	Exposed P, K, Q, E, N, D	Intermediate M, P, S, T, H, Y

**Figure 3:**
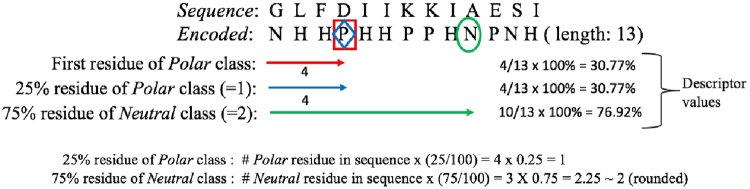
Illustration of the calculations of D_F_ with a sample antibacterial peptide.

We illustrated these computations in Fig. 3 using the sample sequence “GLFDIIKKIAESI” (antibacterial peptide aurein 1.1). According to the hydrophobicity property and its three classes *Polar* (*P*), *Neutral* (*N*), and *Hydrophobic* (*H*), the aurein peptide sequence of length 13 can be rewritten as “NHHPHHPPHNPNH.” The “first residue” of the *Polar* class, which is amino acid D, is the 4th residue in the sequence. Thus, the descriptor value of the “first residue” distribution type of the *Polar* class is computed as (4/13) × 100% = 30.77%. Because there are only four polar residues in this sequence, the first polar residue is also the “25% of class *Polar*” (the same descriptor value is obtained). To compute the “75% residue” distribution type of class *Neutral*, we determine the occurrence $Z\,=\,\lfloor 3\times 0.75\rfloor \,=\,2$, i.e., the 2nd *Neutral* residue. Position *P* of this residue is determined from the sequence, which is the 10th residue, and the descriptor value is then computed as (10/13) × 100% = 76.92%.

With the *Distribution* descriptors, we have five distribution patterns, seven physicochemical properties of amino acids, three classes for each property, thus amounting to 105 descriptors in total for each input sequence.

### Machine learning algorithm

As for the machine learning model, we selected RF^29^ because of its better performance in an initial test against SVM (see Supplementary Table S4). RF has also proven to be successful in numerous bioinformatic applications such as the prediction of phosphorylation sites^30^, DNA-binding proteins^31^, protein–protein interactions^32^, and G protein–coupled receptors^33^. RF employs an ensemble learning approach, which makes predictions by averaging results across many decision trees, and each tree is constructed by random sampling of the training data. The main principle behind ensemble approaches is that a group of learners can come together to form a “strong” learner. Hence, the number of trees is an important parameter for RF methods. In this study, 100 trees were analyzed in all our models because we noticed that the performance was better with 100 trees than with 200 or 500 trees. As the number of input variables tried at each split, commonly known as *mtry*, we adopted the default value, which was the square root of the number of features.

### Cross-validation technique

Performance of a classifier was evaluated by 10-fold cross-validation. In essence, the whole dataset is partitioned into 10 nonoverlapping subsets. In each round, nine subsets are used for training, and one for testing. The process is repeated 10 times to ensure that each subset is utilized once for testing the model that was trained on the other nine.

### Performance metrics

The prediction models were evaluated on sensitivity (*Sn*, also known as recall), *Sp*, *Acc*, *Pr*, and *MCC* with the following definitions:

1 $$Sn=\frac{TP}{TP+FN}$$

2 $$Sp=\frac{TN}{FP+TN}$$

3 $${\Pr }=\frac{TP}{TP+FP}$$

4 $$Acc=\frac{TP+TN}{TP+FN+FP+TN}$$

5 $$MCC=\frac{(TP\times TN)-(FP\times FN)}{\sqrt{(TP+FP)\times (TP+FN)\times (FP+TN)\times (TN+FN)}}$$

where from the confusion matrix, *TP* (true positive) and *TN* (true negative) are correctly predicted positive and negative samples, respectively. Similarly, *FP* (false positive) and *FN* (false negative) are incorrectly predicted positive and negative samples, respectively. Among these measures, *MCC* is the most stringent one; it takes into account both accuracy and error rates of the two classes. *MCC* close to 1.0 means that the classifier has high accuracy and a low misclassification rate.

The prediction models were also evaluated on *AUC-ROC*^34^, *AUC-PR*, and the *κ* statistic^35^. *AUC-ROC* is a popular measure for binary classifiers in machine learning research. It is a two-dimensional curve showing how the number of correctly classified positive samples (*Sn*) varies with the number of incorrectly classified negative samples (1–*Sp*). The AUC of this curve yields a single measure that indicates the robustness of the model, thus making this measure useful. One property of *AUC-ROC* is that it is insensitive to changes in class distribution because the calculation does not couple values from the two classes^34^. Nonetheless, for a prediction task where data for the two classes are highly imbalanced, e.g., the positive interesting samples are small as compared to large negative uninteresting samples, the PrecisionRecall curve will be a better option because both precision and recall focus on analysis of the positively predicted samples. Both *AUC-ROC* and *AUC-PR* return values between 0 and 1; the higher the AUC value, the better is the performance of a classifier.

The *κ* statistic originally served to assess agreement between two raters by comparing the observed agreement versus the hypothetical random agreement in all *N* samples. It is calculated as

6 $$Kappa=\frac{{p}_{o}-{p}_{e}}{1-{p}_{e}}$$

7 $${p}_{e}=\sum _{C}(\frac{{n}_{C}^{A}}{N}\times \frac{{n}_{C}^{B}}{N})$$

where *p*_*o*_ is the probability of observed agreement (i.e., both raters give the same answer) and *p*_*e*_ is the probability of overall random agreement. ${n}_{C}^{A}$ and ${n}_{C}^{B}$ are the total numbers of samples rated as class *C* by raters *A* and *B*, respectively. Therefore, *κ* measures the observed agreement between the two raters and adjusts it for the extent of agreement that could be expected by chance. To assess a classification model with an imbalanced dataset, *κ* can help to estimate how credible the prediction accuracy is by factoring out the number of guess predictions. The higher the value of *κ*, the more reliable is a classification model. With the conventional notations for the confusion matrix, we can express equations for *p*_*o*_ and *p*_*e*_ as follows:

8 $${p}_{o}=\frac{(TP+TN)}{TP+TN+FP+FN}$$

9 $${p}_{e}=\frac{(TP+FN)\times (TP+FP)\times (TN+FN)\times (TN+FP)}{{(TP+TN+FP+FN)}^{2}}$$

The *κ* statistic can range from −1 to 1 though values below 0 are rare. As suggested by Li *et al*., credibility of the prediction accuracy can be classified into three levels: 1) *κ* ≥ 0.75, credibility is high; 2) 0.4 ≤ *κ* < 0.75, credibility is moderate; 3) *κ* < 0.4, credibility is low^15^.

Given that each performance metric above evaluates a different characteristic of the model, here, we propose a combined measure, named as *C-measure*, to facilitate the selection of an optimal model. It is calculated as

10 $$C \mbox{-} measure=AUC \mbox{-} ROC\times AUC \mbox{-} PR\times MCC\times Kappa$$

In all the models tested, we obtained positive values for *MCC* and *κ*; thus, *C-measure* also lies between 0 and 1.

### Implementation

The propy 1.0 package^18^ was employed to calculate descriptor values. Model training and testing were implemented in MATLAB via the TreeBagger function of the Statistics and Machine learning toolbox.

### Data availability

The datasets generated and analyzed in this study can be freely downloaded on the CBBio lab homepage http://cbbio.cis.umac.mo/software/AmPEP. The prediction model in MATLAB code is available upon an e-mail request.

## Electronic supplementary material

Supplementary information
