# Supplementary material for: AmPEP: Sequence-based prediction of antimicrobial peptides using distribution patterns of amino acid properties and random forest
Source: Sci Rep. 2018 Jan 26;8:1697. doi: 10.1038/s41598-018-19752-w (PMC5785966; doi:10.1038/s41598-018-19752-w)

Supplementary Information

AmPEP: Sequence-based prediction of antimicrobial peptides using distribution patterns of amino acid properties and random forest

Pratiti Bhadra, Jielu Yan, Jinyan Li, Simon Fong, and Shirley W. I. Siu*

Department of Computer and Information Science, University of Macau
Avenida da Universidade, Taipa, Macau, China

*Corresponding author: shirleysiu@umac.mo

Last update: 14 Dec 2017

Supplementary Table S1. Pearson correlation coefficients (PCC) of AMP/non-AMP distributions using M^{model_train}. A descriptor is named with its *physiochemical property*, *class*, and *distribution* ("first residue" is coded as *001*, "25% residues" as *025*, "50% residues" as *050*, "75% residues" as *075*, "last residue" as *100*). Descriptors with PCC < 0.5 are shown with boldface; those with PCC < 0.5 also in the two other datasets (C^{train}, C^{test}) are marked with asterisks.

Descriptor	PCC	Descriptor	PCC	Descriptor	PCC
Charge_C1_001	0.690	Polarizability_C3_025	0.751	NormalizedVDWV_C2_075	0.632
**Charge_C2_001***	**0.063**	SecondaryStr_C1_025	0.797	NormalizedVDWV_C3_075	0.675
**Charge_C3_001**	**0.252**	SecondaryStr_C2_025	0.711	Polarity_C1_075	0.730
Hydrophobicity_C1_001	0.747	SecondaryStr_C3_025	0.571	**Polarity_C2_075***	**0.311**
Hydrophobicity_C2_001	0.549	SolventAccessibility_C1_025	0.643	Polarity_C3_075	0.544
**Hydrophobicity_C3_001***	**0.128**	SolventAccessibility_C2_025	0.729	Polarizability_C1_075	0.525
NormalizedVDWV_C1_001	0.554	SolventAccessibility_C3_025	0.669	Polarizability_C2_075	0.801
NormalizedVDWV_C2_001	0.625	Charge_C1_050	0.597	Polarizability_C3_075	0.675
**NormalizedVDWV_C3_001***	**0.301**	**Charge_C2_050***	**0.288**	SecondaryStr_C1_075	0.747
**Polarity_C1_001***	**0.130**	**Charge_C3_050**	**0.268**	SecondaryStr_C2_075	0.807
Polarity_C2_001	0.512	Hydrophobicity_C1_050	0.679	SecondaryStr_C3_075	0.627
Polarity_C3_001	0.689	Hydrophobicity_C2_050	0.719	SolventAccessibility_C1_075	0.727
Polarizability_C1_001	0.584	Hydrophobicity_C3_050	0.683	SolventAccessibility_C2_075	0.609
Polarizability_C2_001	0.614	NormalizedVDWV_C1_050	0.628	SolventAccessibility_C3_075	0.613
**Polarizability_C3_001***	**0.301**	NormalizedVDWV_C2_050	0.627	Charge_C1_100	0.571
**SecondaryStr_C1_001***	**0.346**	NormalizedVDWV_C3_050	0.672	**Charge_C2_100**	**0.484**
SecondaryStr_C2_001	0.657	Polarity_C1_050	0.710	**Charge_C3_100***	**0.184**
SecondaryStr_C3_001	0.768	**Polarity_C2_050***	**0.357**	**Hydrophobicity_C1_100***	**0.464**
**SolventAccessibility_C1_001**	**0.432**	Polarity_C3_050	0.648	Hydrophobicity_C2_100	0.559
SolventAccessibility_C2_001	0.747	Polarizability_C1_050	0.636	Hydrophobicity_C3_100	0.652
**SolventAccessibility_C3_001***	**0.296**	Polarizability_C2_050	0.684	NormalizedVDWV_C1_100	0.529
Charge_C1_025	0.588	Polarizability_C3_050	0.672	NormalizedVDWV_C2_100	0.558
Charge_C2_025	0.661	SecondaryStr_C1_050	0.720	NormalizedVDWV_C3_100	0.567
**Charge_C3_025**	**0.237**	SecondaryStr_C2_050	0.739	Polarity_C1_100	0.645
Hydrophobicity_C1_025	0.729	SecondaryStr_C3_050	0.640	Polarity_C2_100	0.521

Hydrophobicity_C2_025	0.782	**SolventAccessibility_C1_050**	**0.432**	Polarity_C3_100	0.570
Hydrophobicity_C3_025	0.640	SolventAccessibility_C2_050	0.679	Polarizability_C1_100	0.503
NormalizedVDWV_C1_025	0.703	SolventAccessibility_C3_050	0.629	Polarizability_C2_100	0.565
NormalizedVDWV_C2_025	0.617	**Charge_C1_075**	**0.473**	Polarizability_C3_100	0.567
NormalizedVDWV_C3_025	0.751	Charge_C2_075	0.782	**SecondaryStr_C1_100***	**0.420**
Polarity_C1_025	0.636	**Charge_C3_075**	**0.231**	SecondaryStr_C2_100	0.723
**Polarity_C2_025***	**0.315**	Hydrophobicity_C1_075	0.609	SecondaryStr_C3_100	0.618
Polarity_C3_025	0.657	Hydrophobicity_C2_075	0.696	SolventAccessibility_C1_100	0.546
Polarizability_C1_025	0.705	Hydrophobicity_C3_075	0.727	**SolventAccessibility_C2_100***	**0.464**
Polarizability_C2_025	0.731	NormalizedVDWV_C1_075	0.638	SolventAccessibility_C3_100	0.639

Supplementary Table S2. Datasets generated from M^{model_train} for P:N ratio tests of AMP prediction. Size of the positive dataset is 3268.

P:N ratio	Size of one non-AMP subset	Total number of non-AMP subsets
1:1	3268	51
1:1.5	4902	34
1:2	6536	26
1:2.5	8170	20
1:3	9804	17
1:3.5	11438	15
1:4	13072	13
1:4.5	14706	11
1:5	16340	10
1:5.5	17974	9
1:6	19608	9
1:6.5	21242	8
1:7	22876	7
1:7.5	24510	7
1:8	26144	6
1:8.5	27778	6
1:9	29412	6
1:9.5	31046	5
1:10	32680	5

Supplementary Table S3. Performance of RF classifiers using different P:N ratios in 10-fold cross validation. Values shown are averages and standard deviations (in brackets) over all corresponding subsets. The optimal model based on C-measure is ratio 1:3.

P:N ratio	*Sn*	*Sp*	*Acc*	*MCC*	*AUC-ROC*	*AUC-PR*	*Kappa*	*C-measure*
1:1	0.978 (0.002)	0.945 (0.004)	0.962 (0.002)	0.924 (0.004)	0.988 (0.001)	0.698 (0.024)	0.923 (0.005)	0.588 (0.018)
1:1.5	0.972 (0.002)	0.952 (0.003)	0.960 (0.002)	0.917 (0.004)	0.989 (0.001)	0.755 (0.017)	0.917 (0.004)	0.628 (0.011)
1:2	0.965 (0.003)	0.957 (0.002)	0.960 (0.002)	0.912 (0.004)	0.989 (0.001)	0.791 (0.014)	0.911 (0.005)	0.650 (0.009)
1:2.5	0.958 (0.003)	0.961 (0.002)	0.961 (0.002)	0.906 (0.004)	0.989 (0.001)	0.814 (0.015)	0.905 (0.004)	0.660 (0.010)
1:3	0.950 (0.003)	0.965 (0.002)	0.962 (0.002)	0.900 (0.004)	0.989 (0.000)	0.830 (0.009)	0.899 (0.004)	0.665 (0.006)
1:3.5	0.943 (0.004)	0.968 (0.001)	0.962 (0.002)	0.893 (0.005)	0.989 (0.001)	0.840 (0.010)	0.893 (0.005)	0.663 (0.009)
1:4	0.936 (0.004)	0.970 (0.002)	0.963 (0.002)	0.888 (0.005)	0.989 (0.001)	0.849 (0.007)	0.888 (0.005)	0.663 (0.007)
1:4.5	0.929 (0.004)	0.973 (0.001)	0.965 (0.002)	0.884 (0.005)	0.989 (0.000)	0.857 (0.007)	0.884 (0.005)	0.662 (0.008)
1:5	0.921 (0.004)	0.974 (0.001)	0.965 (0.001)	0.878 (0.003)	0.989 (0.001)	0.858 (0.004)	0.877 (0.003)	0.653 (0.005)
1:5.5	0.915 (0.006)	0.975 (0.001)	0.966 (0.001)	0.873 (0.004)	0.989 (0.000)	0.862 (0.006)	0.873 (0.004)	0.649 (0.007)
1:6	0.908 (0.005)	0.977 (0.001)	0.967 (0.001)	0.868 (0.004)	0.989 (0.001)	0.862 (0.007)	0.867 (0.004)	0.642 (0.010)
1:6.5	0.902 (0.006)	0.978 (0.001)	0.968 (0.001)	0.863 (0.006)	0.989 (0.001)	0.864 (0.005)	0.863 (0.006)	0.637 (0.009)
1:7	0.894 (0.004)	0.979 (0.001)	0.968 (0.001)	0.858 (0.004)	0.989 (0.001)	0.864 (0.005)	0.858 (0.004)	0.629 (0.008)
1:7.5	0.889 (0.007)	0.980 (0.001)	0.969 (0.001)	0.854 (0.007)	0.989 (0.001)	0.864 (0.007)	0.854 (0.006)	0.623 (0.011)
1:8	0.882 (0.009)	0.981 (0.001)	0.970 (0.002)	0.850 (0.008)	0.989 (0.001)	0.863 (0.004)	0.850 (0.008)	0.616 (0.013)
1:8.5	0.875 (0.009)	0.982 (0.001)	0.971 (0.001)	0.846 (0.007)	0.989 (0.000)	0.859 (0.005)	0.846 (0.007)	0.608 (0.012)
1:9	0.869 (0.007)	0.982 (0.000)	0.971 (0.001)	0.841 (0.006)	0.989 (0.000)	0.858 (0.006)	0.841 (0.006)	0.601 (0.012)
1:9.5	0.861 (0.005)	0.983 (0.001)	0.972 (0.001)	0.837 (0.006)	0.989 (0.000)	0.857 (0.002)	0.836 (0.006)	0.593 (0.010)
1:10	0.859 (0.006)	0.984 (0.001)	0.972 (0.001)	0.835 (0.005)	0.989 (0.001)	0.857 (0.006)	0.835 (0.005)	0.590 (0.011)

Supplementary Table S4. Comparison of RF and SVM classifiers using D_F features and AMP/non-AMP data ratio of 1:3 in 10-fold cross-validation. Values shown are averages and standard deviations (in brackets) over all corresponding subsets

Method	*Sn*	*Sp*	*Acc*	*MCC*	*AUC-ROC*	*AUC-PR*	*Kappa*	*C-measure*
RF	0.950 (0.003)	0.965 (0.002)	0.962 (0.002)	0.900 (0.004)	0.989 (0.000)	0.830 (0.009)	0.889 (0.004)	0.665 (0.006)
SVM	0.532 (0.042)	0.949 (0.006)	0.844 (0.012)	0.552 (0.038)	0.813 (0.030)	0.681 (0.034)	1.0 (0.000)	0.305 (0.047)

Supplementary Table S5. A comparison of RF classifiers using different descriptors by 10-fold cross-validation with the AMP data ratio of 1:1. Values shown are averages and standard deviations (in brackets) over 10 times of 10-fold cross validation. The best two results in each performance measure are highlighted.

**Feature set {#}**	***Sn***	***Sp***	***Acc***	***MCC***	***AUC-ROC***	***AUC-PR***	***Kappa***	***C-measure***
AmPEP {105}	**0.978** (0.002)	**0.945** (0.004)	**0.962** (0.002)	**0.924** (0.004)	**0.988** (0.001)	0.698 (0.024)	**0.923** (0.005)	0.588 (0.018)
AAC {20}	**0.948** (0.002)	**0.946** (0.001)	**0.947** (0.001)	**0.894** (0.002)	**0.985** (0.000)	0.77 (0.004)	**0.894** (0.002)	0.606 (0.004)
PAAC {24}	0.948 (0.001)	**0.945** (0.002)	**0.947** (0.001)	0.893 (0.001)	0.984 (0.000)	0.822 (0.006)	0.893 (0.001)	**0.645** (0.006)
K-mer {400}	0.939 (0.002)	0.944 (0.002)	0.941 (0.001)	0.883 (0.002)	0.983 (0.000)	**0.876** (0.005)	0.883 (0.002)	**0.671** (0.006)
Auto Covariance (AC) {6}	0.761 (0.002)	0.844 (0.003)	0.802 (0.002)	0.606 (0.004)	0.870 (0.001)	0.814 (0.004)	0.604 (0.004)	0.259 (0.005)
Cross Covariance (CC) {12}	0.802 (0.003)	0.85 (0.003)	0.826 (0.003)	0.653 (0.005)	0.897 (0.002)	0.851 (0.002)	0.652 (0.005)	0.325 (0.005)
Auto-Cross Covariance (ACC) {18}	0.83 (0.002)	0.863 (0.003)	0.846 (0.002)	0.693 (0.004)	0.914 (0.001)	**0.863** (0.003)	0.693 (0.004)	0.379 (0.005)
Parallel Correlation Pseudo Amino Acid Composition (PC-PseAAC) {22}	0.948 (0.001)	**0.945** (0.002)	**0.947** (0.002)	0.893 (0.003)	0.984 (0.000)	0.806 (0.006)	0.893 (0.003)	0.633 (0.007)
Series Correlation Pseudo Amino Acid Composition (SC-PseAAC) {26}	**0.948** (0.001)	**0.946** (0.002)	**0.947** (0.001)	0.893 (0.002)	0.984 (0.000)	0.805 (0.005)	0.893 (0.002)	0.633 (0.006)
General Parallel Correlation Pseudo Amino Acid Composition (PC-PseAAC-General) {22}	0.946 (0.001)	0.942 (0.001)	0.944 (0.001)	0.888 (0.002)	0.984 (0.000)	0.823 (0.006)	0.888 (0.002)	0.639 (0.005)
Parallel Series Correlation Pseudo Amino Acid Composition (SC-PseAAC-General) {26}	0.946 (0.001)	0.943 (0.002)	0.944 (0.001)	0.889 (0.002)	0.983 (0.000)	0.822 (0.005)	0.889 (0.002)	0.639 (0.005)

AAC: Amino Acid Composition, PAAC: Pseudo Amino Acid Composition
AAC and PseAAC were generated using propy 1.0 package (default parameter of propy is used).
Other descriptors, K-mer, AC, CC, ACC, PC-PseAAC, SC-PseAAC, PC-PseAAC-General, SC-PseAAC-General were generated by Pse-in-One-1.0.4 using default parameters.

Supplementary Figure S1. Comparison of the AMP and non-AMP statistical distributions of 105 descriptors.

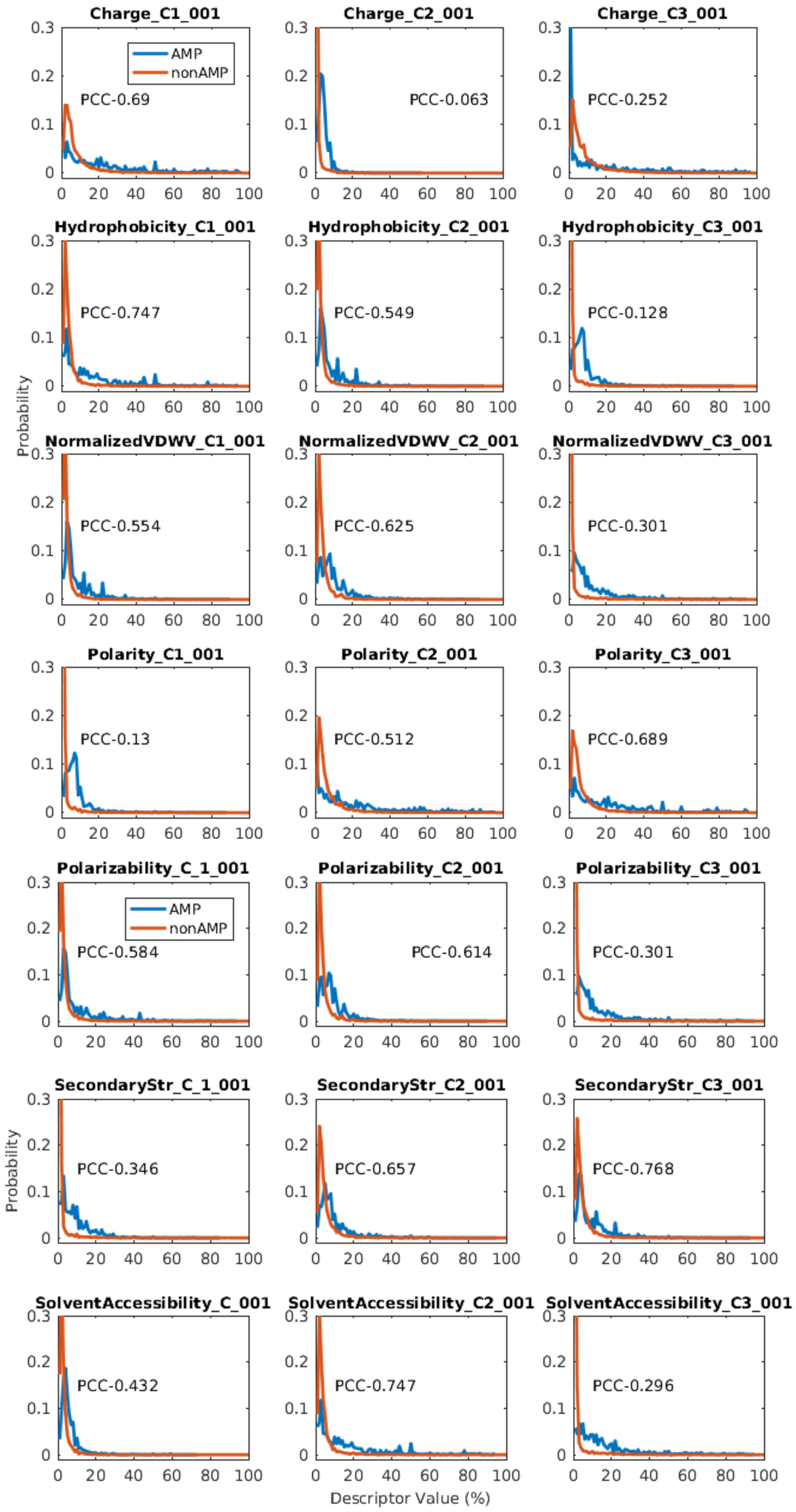

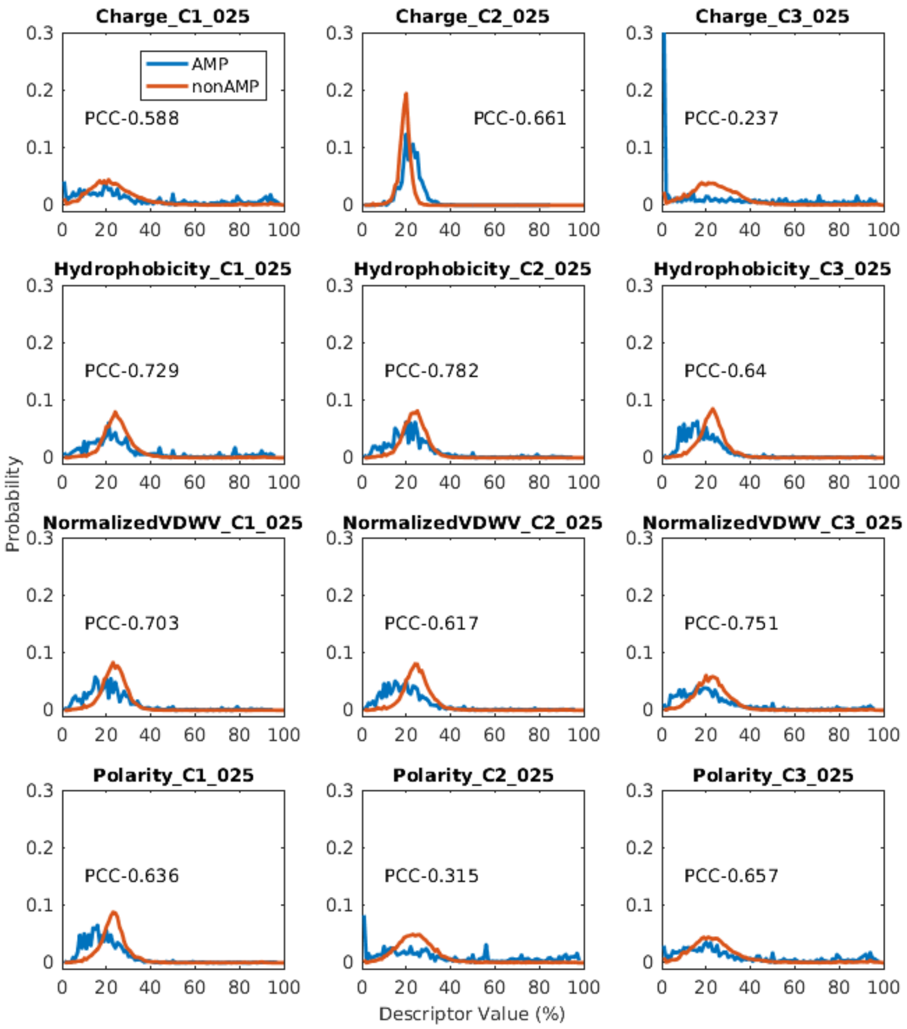
Charge_C1_025
AMP
nonAMP
PCC-0.588
Charge_C2_025
PCC-0.661
Charge_C3_025
PCC-0.237
Hydrophobicity_C1_025
PCC-0.729
Hydrophobicity_C2_025
PCC-0.782
Hydrophobicity_C3_025
PCC-0.64
NormalizedVDWV_C1_025
PCC-0.703
NormalizedVDWV_C2_025
PCC-0.617
NormalizedVDWV_C3_025
PCC-0.751
Polarity_C1_025
PCC-0.636
Polarity_C2_025
PCC-0.315
Polarity_C3_025
PCC-0.657
Probability
Descriptor Value (%)

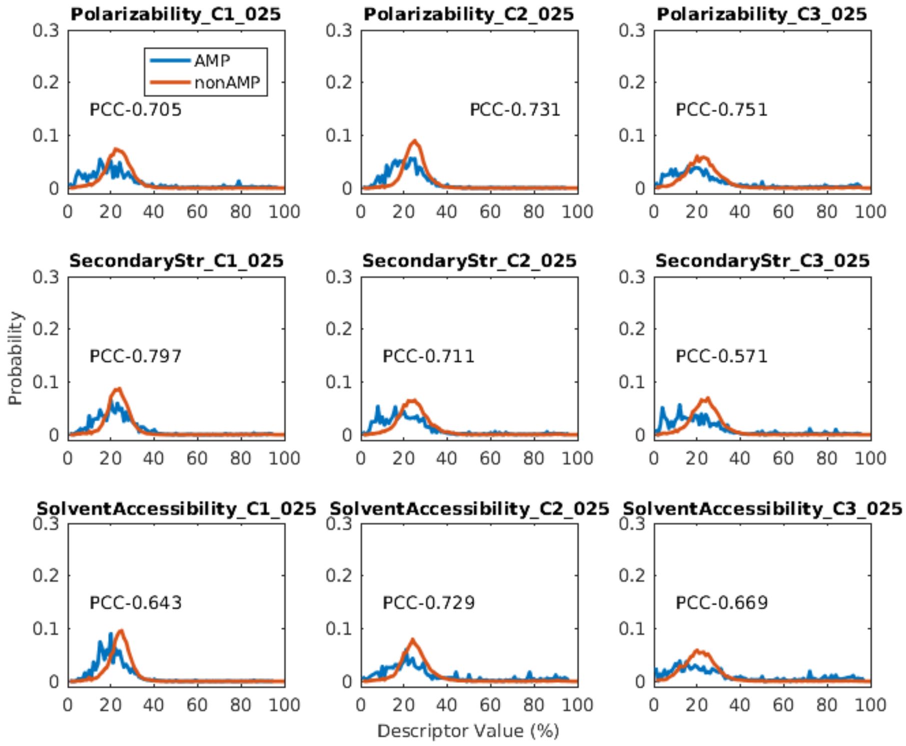
Polarizability_C1_025
AMP
nonAMP
PCC-0.705
Polarizability_C2_025
PCC-0.731
Polarizability_C3_025
PCC-0.751
SecondaryStr_C1_025
PCC-0.797
SecondaryStr_C2_025
PCC-0.711
SecondaryStr_C3_025
PCC-0.571
SolventAccessibility_C1_025
PCC-0.643
SolventAccessibility_C2_025
PCC-0.729
SolventAccessibility_C3_025
PCC-0.669
Probability
Descriptor Value (%)

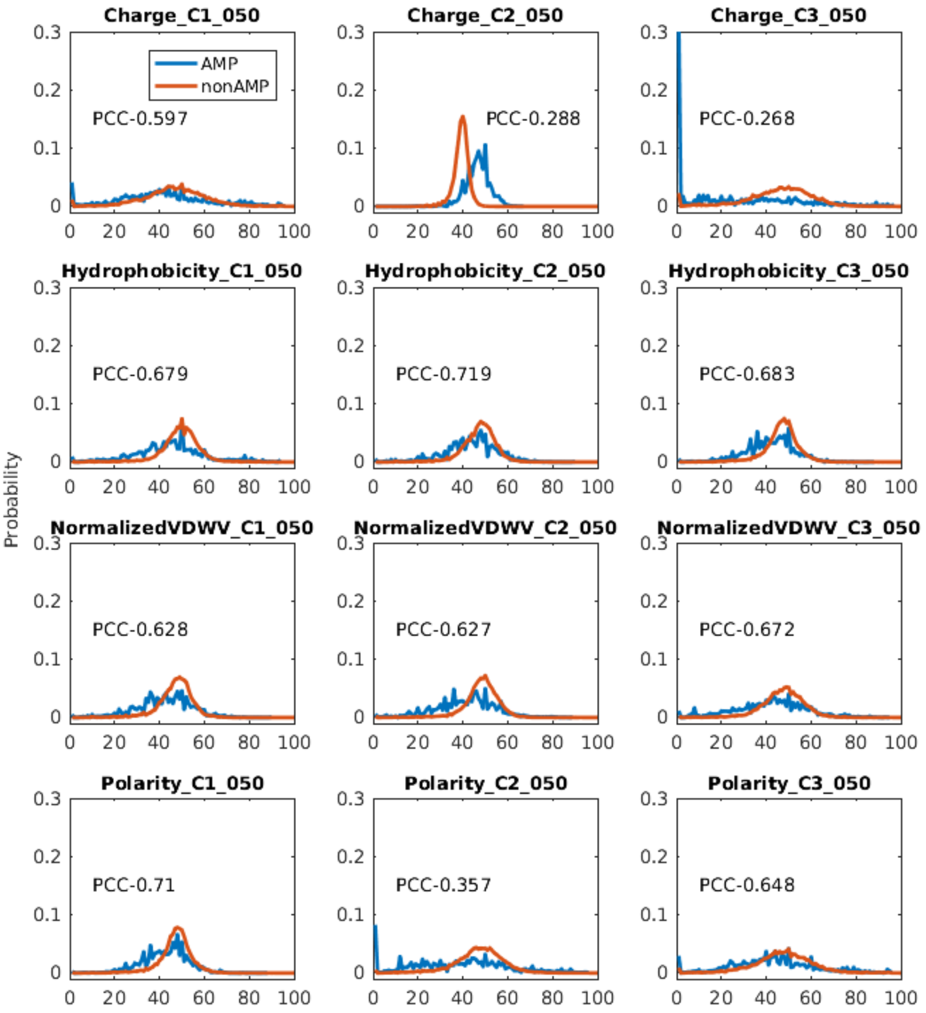
Charge_C1_050
AMP
nonAMP
PCC-0.597
Charge_C2_050
PCC-0.288
Charge_C3_050
PCC-0.268
Hydrophobicity_C1_050
PCC-0.679
Hydrophobicity_C2_050
PCC-0.719
Hydrophobicity_C3_050
PCC-0.683
Probability
NormalizedVDWV_C1_050
PCC-0.628
NormalizedVDWV_C2_050
PCC-0.627
NormalizedVDWV_C3_050
PCC-0.672
Polarity_C1_050
PCC-0.71
Polarity_C2_050
PCC-0.357
Polarity_C3_050
PCC-0.648
0.3
0.2
0.1
0
0 20 40 60 80 100

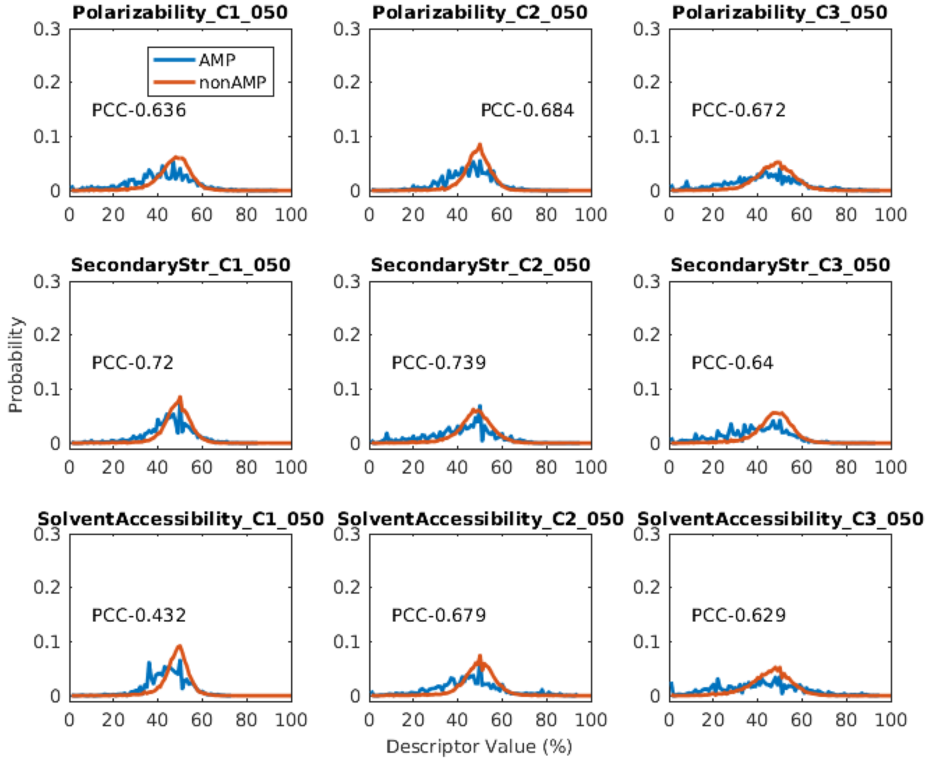
Polarizability_C1_050
AMP
nonAMP
PCC-0.636
Polarizability_C2_050
PCC-0.684
Polarizability_C3_050
PCC-0.672
SecondaryStr_C1_050
PCC-0.72
SecondaryStr_C2_050
PCC-0.739
SecondaryStr_C3_050
PCC-0.64
Probability
SolventAccessibility_C1_050
PCC-0.432
SolventAccessibility_C2_050
PCC-0.679
SolventAccessibility_C3_050
PCC-0.629
0.3
0.2
0.1
0
0 20 40 60 80 100
Descriptor Value (%)

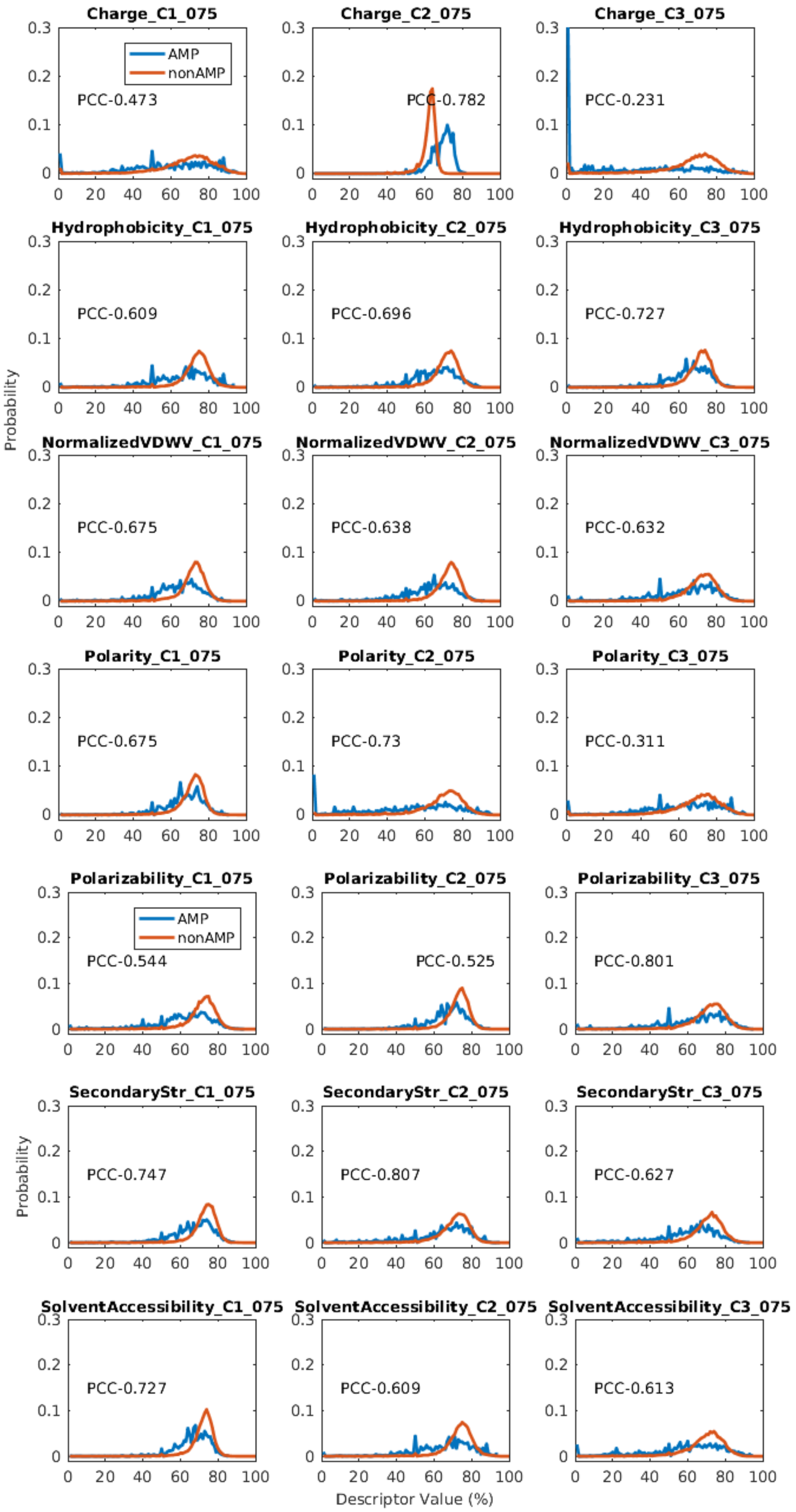
Charge_C1_075
Charge_C2_075
Charge_C3_075
AMP
nonAMP
PCC-0.473
PCC-0.782
PCC-0.231
Hydrophobicity_C1_075
Hydrophobicity_C2_075
Hydrophobicity_C3_075
PCC-0.609
PCC-0.696
PCC-0.727
NormalizedVDWV_C1_075
NormalizedVDWV_C2_075
NormalizedVDWV_C3_075
PCC-0.675
PCC-0.638
PCC-0.632
Polarity_C1_075
Polarity_C2_075
Polarity_C3_075
PCC-0.675
PCC-0.73
PCC-0.311
Polarizability_C1_075
Polarizability_C2_075
Polarizability_C3_075
PCC-0.544
PCC-0.525
PCC-0.801
SecondaryStr_C1_075
SecondaryStr_C2_075
SecondaryStr_C3_075
PCC-0.747
PCC-0.807
PCC-0.627
SolventAccessibility_C1_075
SolventAccessibility_C2_075
SolventAccessibility_C3_075
PCC-0.727
PCC-0.609
PCC-0.613
Probability
Descriptor Value (%)

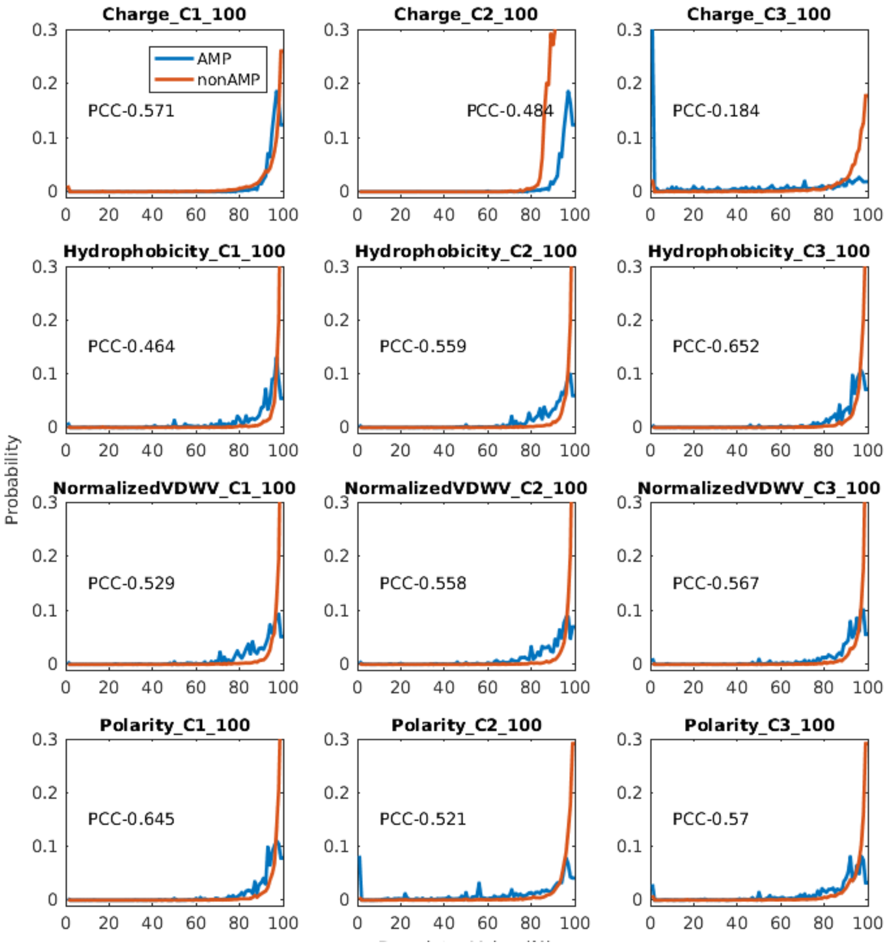
Charge_C1_100
AMP
nonAMP
PCC-0.571
Charge_C2_100
PCC-0.484
Charge_C3_100
PCC-0.184
Hydrophobicity_C1_100
PCC-0.464
Hydrophobicity_C2_100
PCC-0.559
Hydrophobicity_C3_100
PCC-0.652
NormalizedVDWV_C1_100
PCC-0.529
NormalizedVDWV_C2_100
PCC-0.558
NormalizedVDWV_C3_100
PCC-0.567
Polarity_C1_100
PCC-0.645
Polarity_C2_100
PCC-0.521
Polarity_C3_100
PCC-0.57
Probability

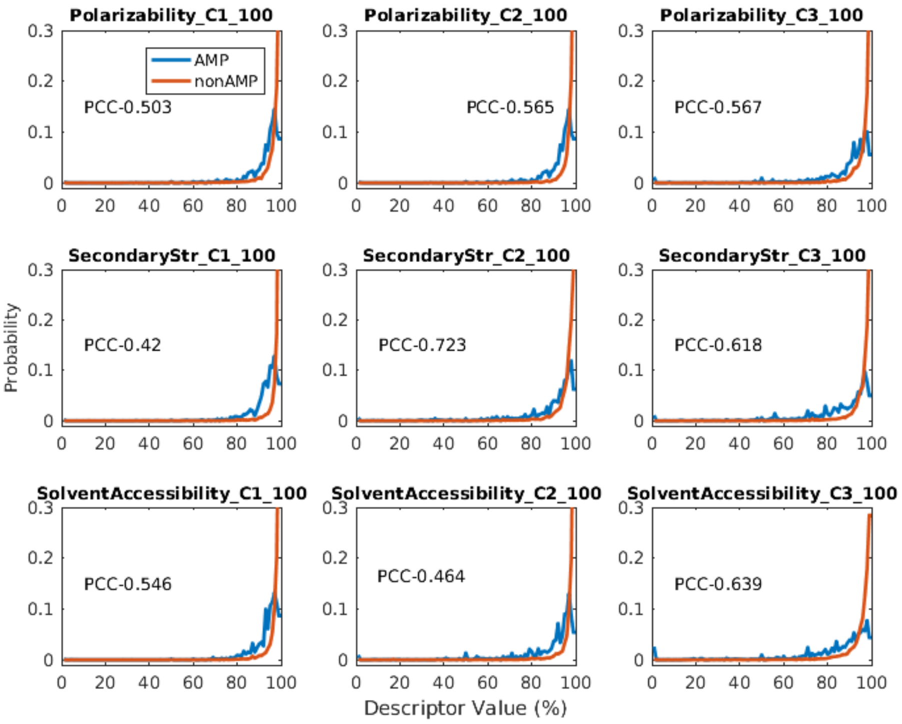
Polarizability_C1_100
AMP
nonAMP
PCC-0.503
Polarizability_C2_100
PCC-0.565
Polarizability_C3_100
PCC-0.567
SecondaryStr_C1_100
PCC-0.42
SecondaryStr_C2_100
PCC-0.723
SecondaryStr_C3_100
PCC-0.618
SolventAccessibility_C1_100
PCC-0.546
SolventAccessibility_C2_100
PCC-0.464
SolventAccessibility_C3_100
PCC-0.639
Probability
Descriptor Value (%)

Supplementary Figure S2. Comparison of the average descriptor value of “first residue” and “100% residues” computed from the AMP sequences of three datasets (M^{model_train}, C^{test} and C^{train}). Standard deviations are shown as error bars.

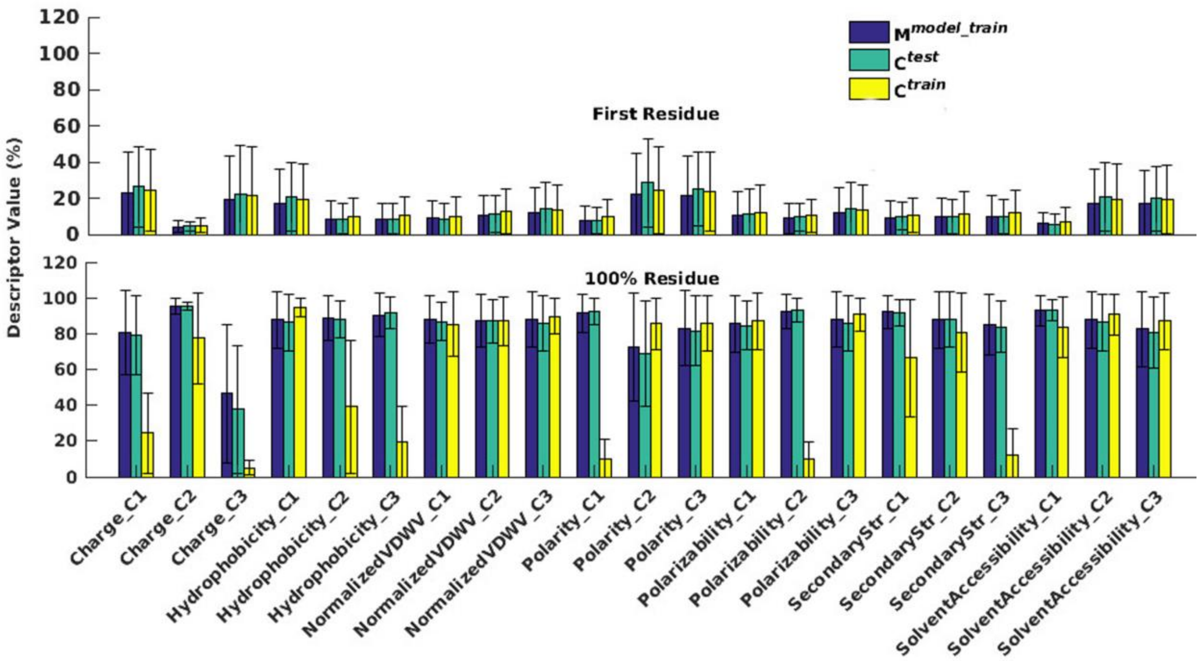

Supplement: Supplementary file 1 — Supplementary information [file 41598_2018_19752_MOESM1_ESM.pdf]
